# Dipentaerythritol penta-acrylate phosphate - an alternative phosphate ester monomer for bonding of methacrylates to zirconia

**DOI:** 10.1038/srep39542

**Published:** 2016-12-21

**Authors:** Ying Chen, Franklin R. Tay, Zhicen Lu, Chen Chen, Mengke Qian, Huaiqin Zhang, Fucong Tian, Haifeng Xie

**Affiliations:** 1 Jiangsu Key Laboratory of Oral Diseases, Department of Prosthodontics, Affiliated Hospital of Stomatology, Nanjing Medical University, Nanjing, China; 2 Department of Endodontics, The Dental College of Georgia, Augusta University, Augusta, GA, USA; 3 Jiangsu Key Laboratory of Oral Diseases, Department of Endodontics, Affiliated Hospital of Stomatology, Nanjing Medical University, Nanjing, China; 4 Department of Cariology and Endodontology, Peking University School and Hospital of Stomatology, Beijing, China

## Abstract

The present work examined the effects of dipentaerythritol penta-acrylate phosphate (PENTA) as an alternative phosphate ester monomer for bonding of methacrylate-based resins to yttria-stabilized tetragonal zirconia polycrystals (Y-TZP) and further investigated the potential bonding mechanism involved. Shear bond strength testing was performed to evaluate the efficacy of experimental PENTA-containing primers (5, 10, 15, 20 or 30 wt% PENTA in acetone) in improving resin-Y-TZP bond strength. Bonding without the use of a PENTA-containing served as the negative control, and a Methacryloyloxidecyl dihydrogenphosphate(MDP)-containing primer was used as the positive control. Inductively coupled plasma-mass spectrometry (ICP-MS), X-ray photoelectron spectroscopy (XPS) and Fourier-transform infrared spectroscopy (FTIR) were used to investigate the potential existence of chemical affinity between PENTA and Y-TZP. Shear bond strengths were significant higher in the 15 and 20 wt% PENTA groups. The ICP-MS, XPS and FTIR data indicated that the P content on the Y-TZP surface increased as the concentration of PENTA increased in the experimental primers, via the formation of Zr–O–P bond. Taken together, the results attest that PENTA improves resin bonding of Y-TZP through chemical reaction with Y-TZP. Increasing the concentration of PENTA augments its binding affinity but not its bonding efficacy with zirconia.

Over the past decade, there has been growing interest in incorporating phosphate ester monomers in surface treatment primers for coupling of methacrylate-based resin composites to yttria-stabilized tetragonal zirconia polycrystals (Y-TZP)^1^,^2^,^3^,^4^,^5^. Bonding of phosphate ester monomers to Y-TZP purportedly involves a silane-like coupling mechanism based on hydroxylation-driven chemistry^1^. Because phosphate ester monomers can also adhere to tooth structures via a predominantly chemical bonding mechanism^6^,^7^,^8^, they are increasingly being used for developing phosphate ester monomer containing primers, adhesives and resin-based cements for luting Y-TZP ceramic restorations to tooth structures.

Phosphate ester monomers such as methacryloyloxidecyl dihydrogenphosphate (MDP), dipentaerythritol penta-acrylate phosphate (PENTA) and phosphorylated methacrylates (MP) have been designed and synthesized by different manufacturers. Among these phosphate ester monomers, MDP is the only resin monomer produced in commercial quantities that demonstrates chemical affinity between methacrylate resin and Y-TZP. Chemoanalytical techniques such as time-of-flight secondary ion mass spectrometry (TOF-SIMS), X-ray photoelectron spectroscopy (XPS) and Fourier-transform infrared spectroscopy (FTIR) have been used for investigating the bonding mechanism between MDP and Y-TZP^9^,^10^,^11^,^12^. Computational chemistry has also been employed to demonstrate the coupling of MDP to tetragonal zirconia crystalline clusters^13^. Although PENTA has been used in adhesives for bonding of methacrylate resins to tooth structures, with bond strengths to dentin comparable with those achieved using MDP-containing adhesives^14^,^15^,^16^,^17^, little is known whether PENTA can bond to Y-TZP, and whether chemical affinity exists between PENTA and Y-TZP.

Phosphate ester monomers, by definition, contain a carbon-carbon double bond and a phosphate group [-OP(=O)(OH)_2_]. Nevertheless, PENTA has a 3-D spatial molecular structure that is distinct from MDP, being a linear molecule; PENTA also contains a shorter main chain with five vinyl groups. Compared with MDP, the steric hindrance introduced by the four additional vinyl groups renders the PENTA molecule more viscous, which may be a potential disadvantage when the molecule approaches a Y-TZP surface to establish chemical coupling. It has been shown that the length of the spacer group in MDP-like molecules affects their chemical interaction with hydroxyapatite and dentin^18^,^19^,^20^,^21^. Because the potential of PENTA in coupling chemically to Y-TZP is unknown, the objectives of the present study were to evaluate the efficacy of five experimental primers containing different concentrations of PENTA on bonding of methacrylate-based resins to Y-TZP, and to identify the potential bonding mechanism involved. The information is valuable for designing novel phosphate ester monomers for chemical coupling to Y-TZP. The null hypotheses tested were: 1) PENTA had no chemical affinity with Y-TZP, and 2) the concentration of PENTA in experimental ceramic primer formulations has no effect on the bonding effectiveness of methacrylate-based resins to Y-TZP.

## Methods

### Experimental PENTA-containing primers

Five experimental visible light-polymerizable PENTA-containing primers (5, 10, 15, 20 or 30 wt% PENTA in acetone) were prepared and designated as 5 P, 10 P, 15 P, 20 P, and 30 P. The compositions of these experimental primers are shown in Table 1.

### Shear bonding strength

One hundred and five ceramic plates (10 × 10 × 2 mm^3^) cut from a machinable Y-TZP blocks (Everest ZS-Ronde, KAVO, Kaltenbach & Voigt GmbH & Co. KG, Bismarckring, Germany) were completely sintered according to the manufacturer’s instructions and then sandblasted with 110 μm alumina particles at 0.3 MPa from a distance of 10 mm for 20 sec, using a sandblasting device (JNBP-2, Jianian Futong Medical Equipment Co., Ltd., Tianjin, China). The sandblasted Y-TZP plates were randomly assigned to 7 groups (n = 15). Each Y-TZP plate was surface-conditioned with one of the experimental PENTA-containing primers (5 P, 10 P, 15 P, 20 P, 30 P). A commercially available MDP-containing primer, Z-Primer Plus (ZPMDP; Bisco, USA), was used as the positive control. A negative control (Ctr) was also included by priming zirconia with a control primer containing only acetone, camphorquinone (CQ) and ethyl-4-dimethylamino benzoate (Edmab) in the primer only. After removing the acetone solvent by blowing with oil-free air for 15 sec, each conditioned Y-TZP plate was light-cured for 10 sec using a light-emitting diode-type curing unit (Elipar Free-Light 2, 3 M ESPE, St. Paul. MN, USA). One hundred and five resin composite cylinders (Valux Plus, 3 M ESPE) were prepared; each cylinder was cemented to a primed Y-TZP surface using a methacrylate resin cement (RelyX Veneer, 3 M ESPE) under a constant load. Excess cement was removed prior to light-curing of the bonded assembly for 40 sec. After water storing at room temperature for 4 weeks, the bonded specimens were subjected to shear bond strength testing using a universal testing machine (Instron Model 3365, Norward, MA, USA) at a crosshead speed of 1.0 mm/min. Shear bond strength values (in MPa) were calculated by dividing the load at fracture (in Newtons) with the bonding interfacial area.

Bond strength data were analyzed using one-way analysis of variance (ANOVA) after validating the normality and homogeneity of variance of the data sets. Post-hoc pairwise comparisons were performed the least square difference statistic. For all analyses, statistical significance was set at α = 0.05.

### PENTA-containing primer-conditioned Y-TZP powders

The two experimental PENTA-containing primers that produced the highest and lowest shear bond strength mean values were selected to be used for investigating the potential existence of a chemical bond between PENTA and Y-TZP.

The Y-TZP blocks were ground to generate 600-mesh powers. The Y-TZP powder was ultrasonically cleaned in acetone for 10 min, and centrifuged for 5 min. The sediments were separated and then heated in a muffle furnace (Multimat C, Dentsply, USA) at 550 °C for 10 min. Clean Y-TZP powders were obtained after repeating the aforementioned treatments for three times to remove organic contaminants.

Clean Y-TZP powders were immersed in one of the two selected experimental PENTA-containing primers in the dark condition for 5 days. The treated powders were ultrasonically cleaned in acetone, centrifuged, separated and dried at 80 °C in order for three times to remove free or loosely-attached PENTA.

### Inductively coupled plasma-mass spectrometry (ICP-MS)

The PENTA-treated Y-TZP powders were analyzed with ICP-MS to determine their phosphorus content. Y-TZP powders (in 0.1 g aliquots) were dispersed by a certain percentage of mixture of nitric acid, sulfuric acid, and hydrofluoric acid, and then were submitted to microwave-assisted digestion (Multiwave 3000, Anton Paar Corp., Austria), using a special program (see Supplementary Information online). Each solution derived from microwave digestion was transferred into a clean polyester vase and diluted up to 50 g with 2% nitric acid (electronic grade, DUKSAM Corp., Korea). An ICP-MS instrument (7500ce, Agilent Corp., Santa Clara, CA, USA) was used to determine the phosphorus content in each solution, using a pre-determined calibration curve that correlates the spectrometry readings with known phosphorus concentrations.

### Characterization of chemical bond

The prepared Y-TZP powders were characterized using X-ray photoelectron spectroscopy (XPS) and Fourier transform-infrared spectroscopy (FT-IR) to examine if a chemical affinity exists between PENTA and Y-TZP. X-ray photoelectron spectroscopy (Escalab 250xi, Thermo Fisher Scientific Inc., Walthpam, MA, USA) was performed with monochromatic Al Kα radiation (1486.6 eV photon energy, energy step size 0.050 eV); the binding energy scale was calibrated with respect to the C 1 s signal at 284.8 eV. Fourier transform-infrared spectroscopy (Nicolet 6700, Thermo Fisher Scientific Inc.) was performed in the transmission mode using the KBr pellet technique Infrared spectra between 4,000-400 cm^−1^ at 4 cm^−1^ resolution were collected using 32 scans.

### Interaction between PENTA and tetragonal-ZrO_2_ crystal

A PENTA molecule model was built and optimized based on molecular information supplied by the manufacturer and PubChem (https://pubchem.ncbi.nlm.nih.gov/). A tetragonal zirconia crystal (t-ZrO_2_) was built according to the authors’ previous publication^10^. Interaction between PENTA and Zr_4_O_8_ cluster were calculated using the Our own N-layered Integrated molecular Orbital + molecular Mechanics (ONIOM) method due to the relatively large system. Density functional theory (DFT) and molecular mechanics (MM) method were applied for high level and low level calculation, respectively. The integral was set at ultrafine to acquire a more accurate outcome. Solvent effect was considered by the integral equation formal polarization continuum model. All calculations concerning geometry optimization and thermodynamics functions were performed using the Gaussian 09 software package (Gaussian, Wallingford, CT, USA).

## Results

### Shear bond strength

**Figure 1:**
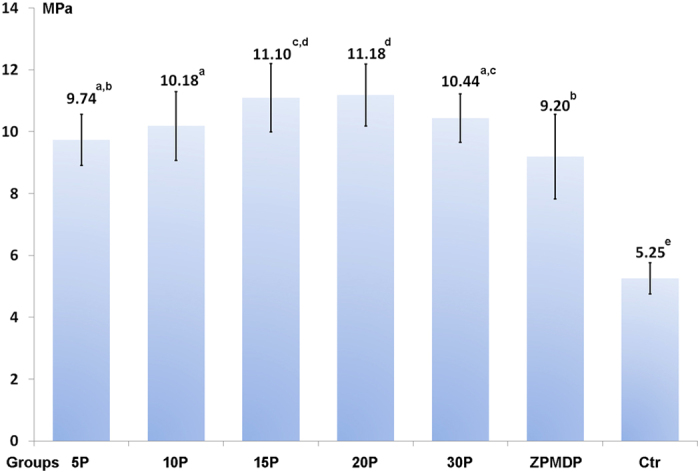
Shear bond strength values of the 5 experimental PENTA-containing ceramic primers, the control MDP-containing ceramic primer, and the phosphate ester monomer-free primer (Ctrl) to Y-TZP. Values are means and standard deviations. Different superscript letters represent group means that were significantly different (p < 0.05).

Parametric statistical analysis was employed because of the normality (Shapiro-Wilk test) and homoscedastic (modified Levene’s test) nature of the data sets. One-way ANOVA indicated the results derived from the five experimental groups and the two control groups were significantly different (F = 63.231, P = 0.000). Pairwise comparisons using the LSD test further indicated shear bond strengths were significant higher in groups 15 P and 20 P, compared with the other groups 5 P, 10 P, 30 P and the positive control, while the negative control group showed the lowest shear bond strength (Fig. 1).

Based on bond strength results, the 20 wt% experimental PENTA-containing primer with the highest shear bond strength value, and the 5 wt% experimental PENTA-containing primer with the lowest shear bond strength mean value were used for ICP-MS, XPS and FTIR.[Table t2][Table t3]

### Chemoanalytic characterization of PENTA-Y-TZP interaction

**Figure 2:**
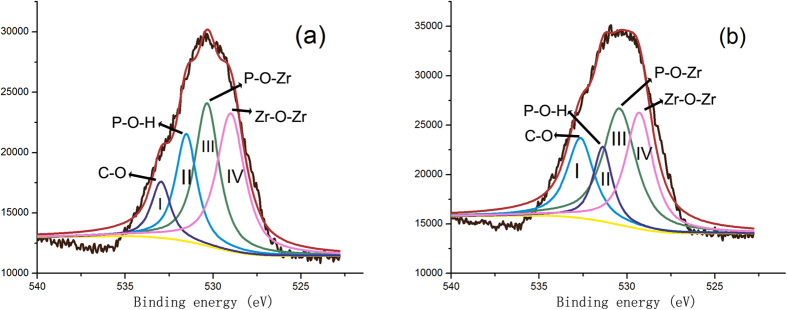
X-ray photoelectron spectroscopy O1s spectra of Y-TZP powders conditioned with experimental primers containing 5 wt% PENTA (**a**) and 20 wt% PENTA (**b**). I (O_C-O_), II (O_P-O-H_), III (O_P-O-Zr_) and IV (O_Zr-O-Zr_) represent the four deconvoluted peaks within the main peak.

**Figure 3:**
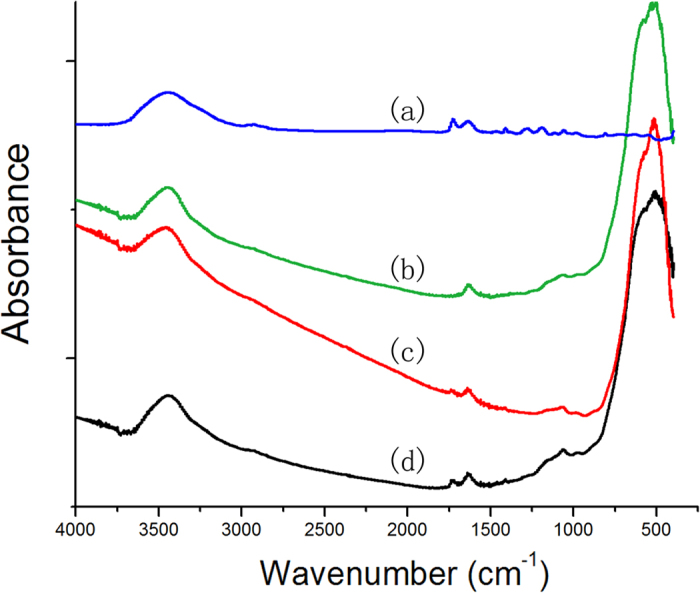
Fourier transform-infrared spectroscopy absorbance spectra of experimental primers containing 5 wt% PENTA (**a**) clean, untreated Y-TZP powder (**b**) and Y-TZP powders conditioned with primer containing 5 wt% PENTA (**c**) and primer containing 20 wt% PENTA (**d**).

- **ICP-MS.** The phosphorus content of Y-TZP powders conditioned with the experimental primers 5 P and 20 P were 374.15 mg/kg and 1293.83 mg/kg, respectively. According to its molecular formula, the molar mass of PENTA is 602 g/mol. Hence, the quantities of bound PENTA (g) on the Y-TZP surface (kg) were 7.27 g/kg and 25.13 g/kg for the two conditioned Y-TZP powders, respectively.
- **XPS.** Figure 2 shows narrow-scan XPS spectra for the O1s region of the two experimental PENTA-containing primer-conditioned Y-TZP powders. Each spectrum was deconvoluted into four contributions identified by peak-fitting procedures. In Fig. 2a (the 5 P primer), the component peak I at a binding energy of 533.0 eV is attributed to the C–O bond^22^. The component peak II at 531.5 eV may be assigned to the P–O–H bond^23^,^24^. Component peak III located at 530.3 eV is mostly attributed to Zr–O–P interactions, but other bonds such as P=O and Zr–O from Zr–OH are also possible^22^,^23^,^24^,^25^. The component peak IV at 529.0 eV represents oxide Zr–O interactions, which suggests the existence of bridging oxygens species, O^2−^ and/or OH^−^, between ZrO_2_ and PENTA^22^,^24^. The percentage area of component peak IV was similar to peak III, which suggests that both ZrO_2_ polycrystal and P–O–Zr bonds are present on the Y-TZP surface. Deconvoluted peaks in Fig. 2b (the 20 P primer) had similar distributions as those present in Fig. 2a. Nevertheless, the small shifts in binding energy suggest possible influence from a higher PENA concentration. Details of the binding energies and relative percentages of the four components, O_C-O_ (I), O_P-O-H_ (II), O_P-O-Zr_ (III), O_Zr-O-Zr_ (IV) are summarized in Table 2. According to atomic percentage of the experimental primers-conditioned Y-TZP powders (Table 3), the phosphorus content was higher in the 20 P primer.
- **FT-IR.** Infrared absorbance spectra of the 5 P primer and Y-TZP powders with and without conditioning are shown in Fig. 3. The strong absorption below 530 cm^−1^ in the spectra of Y-TZP powders is attributed to Zr-O vibrations. The peaks at 3448 cm^−1^ and 1637 cm^−1^ present in the four specimens may be attributed to –OH stretching peaks or physisorbed water^26^. Although the Y-TZP powders conditioned with experimental primers 5 P and 20 P show similar peaks in the fingerprint region, there were minor differences in the stretching peaks of –OH and P-O. The wavenumber of the -OH stretching peak decreased from 3450.62 cm^−1^ and 1637.56 cm^−1^ (5 P) to 3448.11 cm^−1^ and 1637.38 cm^−1^ (20 P), and P-O decreased from 1072.25 cm^−1^ (5 P) to 1061.95 cm^−1^ (20 P).

### Thermodynamic calculations

**Figure 4:**
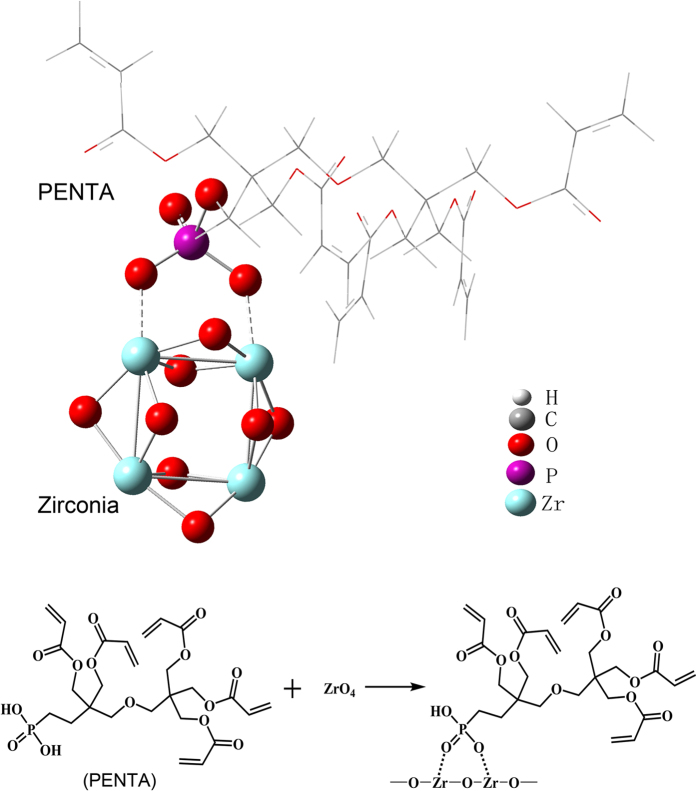
Formation of coordinate bond between PENTA and zirconia via Z-O-P bond based on the computational chemistry model.

The optimized PENTA-t-ZrO_2_ coordination model and corresponding description of atoms are shown in Fig. 4.

The reactions between PENTA and t-ZrO_2_ cluster may be described using the following formula:


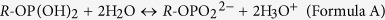



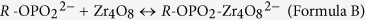


where *R* represents the remaining structure of the PENTA molecule without the phosphate head group.

To study the stability of the configurations between PENTA and t-ZrO_2_, the corresponding Gibbs free energy was calculated, and the thermodynamic data was given in Table 4. All the data were compiled under ambient conditions (1 atm, 298 K). The negative Gibbs free energy suggests that the interaction between PENTA and t-ZrO_2_ proceeds spontaneously on contact.

## Discussion

Although XPS and FT-IR are commonly used to characterize surface chemical bonds, considerable systematic errors and possibly artifacts may be introduced^27^,^28^,^29^,^30^. Each technique, on its own, is inadequate for demonstrating the existence of a chemical bond between the phosphate ester monomer and zirconia. One characteristic of the molecule PENTA is the presence of the element phosphorus, which is absent from untreated Y-TZP and other components of the experimental primers employed in the present study. This enabled the authors to estimate the quantity of PENTA that reacted with Y-TZP by determining the quantity of phosphorus that is bound on the Y-TZP surface after the latter was conditioned with the PENTA-containing experimental primers. Because the amount of PENTA that binds chemically to the Y-TZP surface is miniscule relative to the weight of Y-TZP, a characterization method with high sensitivity and low detection limit, such as ICP-MS, should be employed. In order to exclude the influence of residual phosphorus derived from loosely-attached or free PENTA on the surface of Y-TZP, ultrasonic cleaning with acetone was used repeatedly to remove the aforementioned components, because chemically-bound PENTA could not be removed by acetone. Accordingly, identification of phosphorus in the ICP-MS results confirmed that PENTA is involved in chemical bonding on the surface of Y-TZP. Based on the ICP-MS results, the quantity of phosphorus present in the 20 P primer-conditioned Y-TZP surface was much higher that the quantity present in the 5 P primer-conditioned Y-TZP surface. The data implies that increasing the concentration of PENTA in the experimental primers result in more chemically-bound PENTA on the surface of the Y-TZP.

The XPS spectral data confirms existence of Zr–O–P bonds in both the primer-conditioned Y-TZP specimens. The lower Zr/P ratio and the higher relative presence of O_P-O-Zr_ on the surface of the 20 P primer-conditioned Y-TZP provide additional evidence that the content of bound phosphorus on the Y-TZP surface increased when more PENTA was incorporated in the experimental primers, with the formation of more Zr–O–P bonds. Results of the FT-IR analysis were complementary with the XPS analysis; the presence of more intensive transmittance peaks associated with the –OH and P-O stretching in the 20 P primer-conditioned Y-TZP powder is indicative of more PENTA participating in chemical bonding. Admittedly, the exact nature of the chemical bond between PENTA and Y-TZP cannot be determined using XPS and FT-IR analysis alone. Nevertheless, when those data are examined together with the ICP-MS results and thermodynamic calculations, the qualitative deductions from XPS and FTIR become more convincing. Thermodynamic calculations indicate that the reaction between PENTA and zirconia crystals confirms existence the formation of Zr–O–P bond. Here, the first null hypothesis that “PENTA had no chemical affinity with Y-TZP” has to be rejected. The implication is that PENTA may be used to replace MDP in ceramic primers for bonding of methacrylate resins to zirconia. Based on the chemoanalytical results, chemical bonding between PENTA and Y-TZP is due to the presence of the phosphate group in PENTA. The bonding mechanism should be similar to the formation of chemical bonds between Y-TZP and the well-established phosphate ester monomer MDP. That is, hydroxyls within the phosphate groups of PENTA form coordinate bonds with zirconia crystals.

Although reaction speed is not influenced by the concentration of reactants, the ICP-MS, XPS and FTIR data indicate that increasing the PENTA concentration in the experimental primers results in the more profuse formation of Zr–O–P bonds. For the application of MDP to calcium-containing dental hard tissues, it has been shown that the amount of MDP-Ca salt produced is proportional to the concentration of MDP present in the primer solution, which, in turn, affects the bond strengths of MDP-primed enamel or dentin^31^,^32^,^33^. Hence, the authors speculate that shear bond strength of methacrylate resin composites to PENTA-primed Y-TZP may be affected by the concentration of PENTA present in the experimental ceramic primer solutions. Indeed, the experimental results indicate that primers containing 15 wt% and 20 wt% PENTA achieved the highest shear bond strength with Y-TZP. However, the 20 wt% PENTA group did not provide further improvement in bond strength, and that incorporation of 30 wt% PENTA in the primer resulted in lower bond strength. Based on these results, the second null hypothesis that “the concentration of PENTA in experimental ceramic primer formulations has no effect on the bonding effectiveness of methacrylate-based resins to Y-TZP” has to be rejected. A plausible reason for the decline in bond strength in the 30 wt% PENTA primer is the potential effect of steric hindrance. When the concentration of PENTA is beyond a certain limit, the fluidity and dispersity of the molecule in the primer are reduced. The implication is that there is an optimal range in which PENTA may be incorporated in ceramic primers for improving bonding of methacrylate resins to zirconia.

## Conclusions

The introduction of MDP inflates the development of phosphate ester monomers. Insight into their bonding mechanism is necessary for designing and developing novel phosphate ester monomers with stronger bonding improvement abilities, however, a major problem faced is that knowledge about various developed phosphate ester monomers are quite limited. The present work examined the effects of PENTA as an alternative phosphate ester monomer for bonding of methacrylate-based resins to Y-TZP, and the potential bonding mechanism involved. Based on the present study, and within its limitations, the following conclusions may be drawn:

- PENTA improved the resin bonding of Y-TZP through chemical combined with zirconia;
- Increasing the concentration of PENTA augments its binding affinity with zirconia but not necessarily its bonding efficacy.

## Additional Information

**How to cite this article**: Chen, Y. *et al*. Dipentaerythritol penta-acrylate phosphate - an alternative phosphate ester monomer for bonding of methacrylates to zirconia. *Sci. Rep.* **6**, 39542; doi: 10.1038/srep39542 (2016).

**Publisher's note:** Springer Nature remains neutral with regard to jurisdictional claims in published maps and institutional affiliations.

## Supplementary Material

Supplementary Information

## Figures and Tables

**Table 1:** Compositions of the PENTA-containing experimental primers employed in the present study.

Group	PENTA (wt%)	Acetone (wt%)	CQ (wt%)	EDMAB (wt%)
5 P	5%	93.8%	0.3%	0.9%
10 P	10%	88.8%	0.3%	0.9%
15 P	15%	83.8%	0.3%	0.9%
20 P	20%	78.8%	0.3%	0.9%
30 P	30%	68.8%	0.3%	0.9%

Abbreviations. CQ: camphorquinone; EDMAB, ethyl-4-dimethylamino benzoate.

**Table 2 t2:** Binding energies and relative percentages of the four deconvoluted peaks in the XPS spectra of PENTA-treated Y-TZP powder: O_C-O_ (I), O_P-O-H_ (II), O_P-O-Zr_ (III) and O_Zr-O-Zr_ (IV).

Groups	Bonding energy (eV)				O_1s_ component (%)*			
	I	II	III	IV	I	II	III	IV
5 P	533.0	531.5	530.3	529	10.10	21.52	32.15	36.23
20 P	532.7	531.4	530.4	529.3	20.21	13.54	36.12	30.13

^*^Percentage of each peak was calculated from the relative peak area in the XPS O1s spectrum.

**Table 3 t3:** Atomic percentages of the experimental primers 5P- and 20P-conditioned Y-TZP powders.

Groups	C	O	P	Zr	Zr/P	O/Zr	C/Zr
5 P	30.34	48.71	1.57	19.37	12.34	2.52	1.57
20 P	32.92	46.67	2.51	17.9	7.13	2.60	1.84

**Table 4:** Thermodynamic data of configurations between PENTA and t-ZrO_2_.

	Formula A				Formula B		
	R-OP(OH)_2_	H_2_O	R-OPO_2_^2−^	H_3_O^+^	R-OPO_2_^2−^	ZrO_2_	[R-OPO_2_-ZrO_2_]^2−^
ν	−1	−2	1	2	−1	−1	1
ε_0_+H_corr_(Ha)	−643.522	−75.995	−642.627	−76.384	−642.274	−788.833	−1433.313
S_electron_(Cal/Mol-K)	0	0	0	0	0	0	0
S_rot_(Cal/Mol-K)	38.780	10.317	38.828	9.656	38.805	30.671	41.107
S_vib_(Cal/Mol-K)	176.363	0.004	169.950	1.204	176.467	44.440	213.692
S_tot_(Cal/Mol-K)	215.143	10.321	208.778	10.860	215.272	75.111	254.799
G_final_(Ha)	−643.646	−76.016	−642.748	−76.406	−642.398	−788.887	−1431.456
ΔG(KJ/mol)	309.221				−445.852		

ν: stoichiometric coefficient; ε0: zero-point vibrational energy; H_corr_: thermal corrected enthalpy; Ha: Hartree; S_electron_: entropy of an electron; S_rot_: rotational component of total entropy; S_vib_: vibrational component of total entropy; S_tot_: total entropy; G_fina_l: final Gibbs energy; ΔG: change in free energy.
