# Supplementary material for: Dipentaerythritol penta-acrylate phosphate - an alternative phosphate ester monomer for bonding of methacrylates to zirconia
Source: Sci Rep. 2016 Dec 21;6:39542. doi: 10.1038/srep39542 (PMC5175125; doi:10.1038/srep39542)
Supplement: Supplementary Information [file srep39542-s1.pdf]

Dipentaerythritol penta-acrylate phosphate - an alternative phosphate ester monomer for bonding of methacrylates to zirconia

Ying Chen[1], Franklin R. Tay[2], Zhicen Lu[1], Chen Chen[3], Mengke Qian[1], Huaiqin Zhang[1], Fucong Tian[4], Haifeng Xie[1]*

[1]Jiangsu Key Laboratory of Oral Diseases; Department of Prosthodontics, Affiliated Hospital of Stomatology, Nanjing Medical University, Nanjing, China

[2]Department of Endodontics, The Dental College of Georgia, Augusta University, Augusta, GA, USA

[3]Jiangsu Key Laboratory of Oral Diseases; Department of Endodontics, Affiliated Hospital of Stomatology, Nanjing Medical University, Nanjing, China

[4]Department of Cariology and Endodontology, Peking University School and Hospital of Stomatology, Beijing, China

***Corresponding Author:** Haifeng Xie, M.D.,

Han-Zhong Road 136th,

Stomatological Hospital of Jiangsu Province, Nanjing 210029, China.

Telephone: +8625 8503 1831;

Fax: +8625 8651 6414;

E-mail: xhf-1980@126.com.

Microwave-assisted digestion of ICP-MS analysis

Y-TZP powders (in 0.1 g aliquots) were placed in a polytetrafluoroethylene digestion tank together with 4 mL nitric acid (68.8%~69.8%, electronic grade, DUKSAM Corp., Korea), 2 mL sulfuric acid (electronic grade, DUKSAM Corp., Korea), and 2 mL hydrofluoric acid (MOS level, Beijing Chemical Reagent Research Institute of China, PR China). Microwave-assisted digestion was performed after sealing of the mixture (Multiwave 3000, Anton Paar Corp., Austria), using a special program (Step I: microwave power 800 W, 5 ramps/min, 190 ℃, hold for 5 min; Step II: microwave power 1400 W, 5 ramps/min, 190 ℃, hold for 20 min; Step III; microwave power 0 W, 0 ramp/min, 190 ℃, and hold for 15 min).
